# IL-33 Ameliorates the Development of MSU-Induced Inflammation Through Expanding MDSCs-Like Cells

**DOI:** 10.3389/fendo.2019.00036

**Published:** 2019-02-26

**Authors:** Ke Shang, Yingying Wei, Qun Su, Bing Yu, Ying Tao, Yan He, Youlian Wang, Guixiu Shi, Lihua Duan

**Affiliations:** ^1^Department of Rheumatology and Clinical Immunology, Jiangxi Provincial People's Hospital, Nanchang, China; ^2^Department of Rheumatology and Clinical Immunology, The First Affiliated Hospital of Xiamen University, Xiamen, China

**Keywords:** gout, MDSCs, IL-33, MSU, IL-1β

## Abstract

Interleukin-33 (IL-33), a member of the IL-1 superfamily, has been shown to play a critical role in many diseases through regulating the immune cell responses, including myeloid-derived suppressor cells (MDSCs). Our previous study demonstrated that IL-33 might play a protective role in kidney injury in gout patients by regulating the lipid metabolism. However, the role of IL-33in the development of MSU-induced inflammation remains elusive. In this study, an increased IL-33 expression was observed in gout patients, which was positively correlated with inflammatory marker CRP. To explore the effects and mechanisms of the increased IL-33 expression in the gout patients, the anti-ST2 antibody and exogenous recombinant IL-33 were used in MSU-induced peritonitis animal model that mimics human gout. Compared with control group, mice with exogenous recombinant IL-33 significantly ameliorated the inflammatory cells infiltration, while blockage of IL-33 signaling by anti-ST2 had no effect on the development of MSU-induced peritonitis. Furthermore, the crucial inflammatory cytokine IL-1β was markedly decreased in IL-33-treated mice. Besides that, a large number of anti-inflammatory MDSCs with CD11b^+^Gr1^int^F4/80^+^ phenotype was observed in the IL-33-treated mice, and adoptive transfer of IL-33-induced MDSCs (CD11b^+^Gr1^int^F4/80^+^) markedly inhibited the IL-1β production in MSU-induced peritonitis. In conclusion, our data provide clear evidences that the increased expression of IL-33 in the gout patients might be due to a cause of self-negative regulation, which inhibits the development of MSU-induced inflammation through expanding MDSCs. Thus, IL-33 might serve as a promising therapeutic target for gout.

## Introduction

Gout is the most common inflammatory arthritis caused by inflammatory responses to the deposition of monosodium urate (MSU) crystals. The uric acid levels exceed the physiological saturation concentration, which will lead to monosodium urate crystal (MSU) formation (1). MSU is precipitated in a single crystalline form and deposited in synovial, cartilage, and other tissues around the joint. MSU crystals trigger an intense inflammatory response by activating the resident tissue macrophages and promoting the recruitment of neutrophils to the joint (2). IL-1β is the most important pro-inflammatory regulatory cytokine from activated macrophage by MSU stimulation in the onset of acute gout (3). Likewise, it has also demonstrated that a high level of IL-1β can be detected in the sera of gout patients. During the development of MSU-induced inflammation, mature IL-1β production by macrophages induces activation of IL-l signaling pathways and medullary differentiation factor (MyD88)-dependent NF-κB pathway, resulting in a large number of chemokines and pro-inflammatory factors being produced, and inflammatory immune cells infiltration (4). An interesting character of acute gout is the self-limiting nature of the inflammatory flare. In the absence of clinical intervention, a gouty episode can spontaneously resolve within 7–10 days. However, the mechanism of inflammation self-resolution is still elusive (5, 6).

Interleukin-33 (IL-33), a member of the IL-1 family, is widely expressed in a variety of tissue cells, especially epithelial cells, endothelial cells, and fibroblast cells (7, 8). Numerous studies have shown the complex biological effects of IL-33 in human diseases (9). As an inflammatory factor, IL-33 can promote the pathological development of diseases like asthma, allergic rhinitis and rheumatoid arthritis by inducing type2-immune response and activating mast cells (10–13). In contrast, IL-33 also can act as an anti-inflammatory factor to suppress inflammation by promoting alternatively activated macrophage polarization and Tregs differentiation. Recent studies have shown that IL-33 prevents the development of parasitic infection, allogeneic allograft rejection and atherosclerosis (14, 15). However, the role of IL-33 in the development of MSU-induced inflammation remains unclear.

Myeloid-derived suppressor cells (MDSCs) are a group of heterogeneous cells discovered in recent years, including immature myeloid cells, immature granulocytes, monocytes-macrophages, dendritic cells, and myeloid precursor cells. MDSCs are commonly divided granular cell-like MDSC (CD11b^+^Gr-1^high^) and mononuclear cell-like MDSC (CD11b^+^Gr-1^int^) (16, 17). Numerous studies have shown that MDSCs play a role in the regulation of effector T cells by nitric oxide production, arginase I synthesis and reactive oxygen species (ROS) production, and promoting Treg cells expansion through indoleamine-2,3-dioxygenase (IDO) production (18). In addition, MDSCs also regulate macrophages and dendritic cells function by the production of IL-10 and TGF-β (16). It is known that varieties of factors could affect the induction and activation of MDSCs, such as IL-6, IL-4 and IL-13 (17, 19). In allogeneic transplantation and tumor environments, it has been shown that IL-33 can induce MDSCs expansion, which inhibits transplant rejection and promotes the progression of mouse breast cancer through inhibition of T cell responses (20–22). Recent literature reported that LPS can induce mononuclear cell-like MDSC, which can block the action of neutrophils causing tissue damage by phagocytose them, thereby alleviating bacterial septic shock (23).

In our study, we found that the expression of IL-33 in gout patients was significantly higher than that of the healthy control group, which was positively correlated with inflammatory indicator C-reactive protein (CRP). However, mice treated with exogenous recombinant IL-33 could significantly alleviate MSU-induced inflammation through expanding CD11b^+^Gr1^int^F4/80^+^ MDSCs. However, blockage of endogenous IL-33 signaling by anti-ST2 had no effect on the development of MSU-induced peritonitis. Taken together, our data demonstrated that the increased levels of IL-33 may be a cause of self-negative regulation which inhibits the development of MSU-induced inflammation, and IL-33 might offer an alternative therapy to our current approaches of managing gout.

## Materials and Methods

### Human Subjects and Animals

Fifty-two cases of acute gout patients were collected from the First Affiliated Hospital of Xiamen University in accordance with the diagnosis criteria of American Rheumatology Association &&(1977). Fifty-eighty healthy controls who matched their age and sex were also collected. The research program has been approved by the Ethics Committee of Xiamen University, and all subjects have signed an informed consent form in accordance with the Declaration of Helsinki. Male 6–8 week C57BL/6 were obtained from the Animal Laboratory Center of Xiamen University. All animal studies were conducted in accordance with the guidelines of the Animal Care and use committee of Xiamen University.

### Culture of Human Synovial Fibroblasts

In sterile conditions, we isolated the synovial fibroblasts from the joint fluid of the acute gout patients. Cells were cultured in Dulbecco's modified Eagle's medium plus 10% Fetal Bovine Serum (Hyclone, United States) and seeded at the flask, fed after 48 and 72 h. When the cells grown to sub-confluence (85%) of the culture dishes, used trypsin (Hyclone, United States) to passage cell, rinsed with PBS and plated into the dish. We then collected non-adherent cells after 2 h culture in Dulbecco's modified Eagle's medium plus 10% Fetal Bovine Serum. Repeated passages to the third generation, fibroblast cells can be used for subsequent experiments.

### Histological and Immunohistochemical Analysis

The synovial fibroblasts were put on cover slides in the 6-well plate and were treated with MSU, TNFα, or IL-1β for 24 h. Next, the cell slides were taken out for immunohistochemistry analysis. Cell slides were fixed for 5 min in 4% paraformaldehyde solution, rinsed twice with PBS, followed by 0.5% Triton-100 solution treatment. The slides were treated with 3% BSA (Sangon Biotech, Shanghai) for 30 min at 37°C for blocking non-specific staining. After that, the slides were then incubated with goat anti-human IL-33 Ab (Minneapolis, MN, United States) or control gout IgG at 4° overnight, and were then used hypersensitive two-step immunohistochemical detection reagent (ZSGB-BIO, China) to detect the IL-33 expression levels by microscope.

### Establishment of Acute Gout Animal Model

Male C57BL/6 mice (6–8 weeks old) were grouped into PBS, MSU, IL-33, and IL-33 plus MSU. The mice of PBS group and MSU group were daily administered an intraperitoneal (IP) injection of PBS for 4 days; the mice of IL-33 group and IL-33 + MSU group were daily administered an intraperitoneal (IP) injection of 2 μg rIL-33 for 4 days. On the fourth day, the mice of MSU group and IL-33 plus MSU group were administered an intraperitoneal (IP) injection of 3 mg MSU after inoculation; the mice of PBS group and IL-33 group were administered an intraperitoneal (IP) injection of PBS after inoculation. Expression and purification of mouse recombinant IL-33 were carried out as previously described (24). For blockage of endogenous IL-33 activity, a neutralizing anti-ST2 antibody (DIH4; Biolegend, San Diego, CA, United States) or control IgG (200 μg/mouse) was given into MSU-treated mice 1 h before MSU administration. At 16 h after MSU stimulation, peritoneal exudate cells were harvested by lavage with 3 ml PBS. Cells were analyzed by flow cytometry and lavage fluids were retained for cytokine assay. In the MDSCs adoptive transfer experiments, the CD11b^+^Gr-1^high^F4/80^+^, CD11b^+^Gr-1^int^F4/80^+^, and CD11b^+^Gr-1^int^F4/80^−^ were isolated from IL-33-treated mice, and then the 3 mg MSU per mouse were administered, the lavage fluids were harvested for cytokine assay after 16 h.

### Flow Cytometry

1 × 10^6^ peritoneal exudate cells were obtained, and the cells were incubated with the fluorescent-conjugated monoclonal antibodies in the staining buffer. Antibodies used for flow cytometry are as follows: FITC anti-mouse F4/80, Percy5.5 anti-mouse CD11b and PE/Cy7 anti-mouse Gr-1. All antibodies are purchased from Biolegend (San Diego, CA, United States).

### ELISA (Enzyme-Linked Immunosorbent Assay)

The gout patients and healthy volunteers were collected 3~5 ml through elbow vein in the morning, and put into non-anticoagulant test tube, the serum samples were separated in 2 h. The concentration of IL-33 was determined by ELISA Kit according to the Manufacturer's instruction. IL-1β, IL-6, IL-10, IL-5, and IL-13 in the peritoneal lavage fluid of model mice were also determined by ELISA Kit. All kits were purchased from R&D (Minneapolis, MN, United States).

### Quantitative Real-Time Reverse Transcriptase-Polymerase Chain Reaction

The total RNA was extracted from the collected human PBMC and mouse PECs by TRIzol lysis (Invitrogen) and used Reverse transcription kit (Roche) to acquire cDNA according to the manufacturer's protocol. Then the outcomes were used to analyze the expression level of the target gene arginine1. The sequences were used for the amplification of cDNA derived from mRNA transcripts of the arginine1 gene as shown below: (Forward: 5′-TTG GCA ATT GGA AGC ATC TCT GGC-3′; Reverse: 5′-TCC ACT TGT GGT TGT CAG TGG AGT-3′), and the primer sequences for β-actin gene (Forward: 5′-AGA AAA TCT GGC ACC ACA CC-3′; Reverse: 5′-AGA GGC GTA CAG GGA TAG CA-3′). The sequences were used for the amplification of cDNA derived from mouse PECs mRNA transcripts as shown below: mouse β-actin gene (Forward: 5′-ACC TTC TAC AAT GAG CTG CG-3′; Reverse: 5′-CTG GAT GGC TAC GTA CAT GG-3′), mouse IL-1β gene (Forward: 5′-ACG GAC CCC AAA AGA TGA AG-3′; Reverse: 5′-TTC TCC ACA GCC ACA ATG AG-3′), mouse IL-6 gene (Forward: 5′-AAA CCG CTA TGA AGT TCC TCT C-3′; Reverse: 5′-GTG GTA TCC TCT GTG AAG TCT C-3′), mouse nlrp3 gene (Forward: 5′-ACC TTT GCC CAT ACC TTC AG-3′; Reverse: 5′-TGC CAC AAA CCT TCC ATC TAG-3′), mouse caspase-1 gene (Forward: 5′-TCT GTA TTC ACG CCC TGT TG-3′; Reverse: 5′-GAT AAA TTG CTT CCT CTT TGC CC-3′). The obtained data were analyzed on the ABI7500. Relative expression levels for cytokines were normalized by β-actin and calculated by using the 2^−ΔΔCt^ method.

### Statistical Analysis

Data are presented as mean ± SEM. Group comparisons were performed using Student's *t*-test by GraphPad Prism software; *p*-values (two-tailed) below 0.05 were considered as significant. The Mann-Whitney *U-*test and Spearman's correlation analysis were used to calculate the clinical sample significance. Statistical significance was accepted for *p* < 0.05.

## Results

### Positive Correlation of Increased Serum IL-33 With Disease Activity Index CRP in Gout Patients

**Figure 1:**
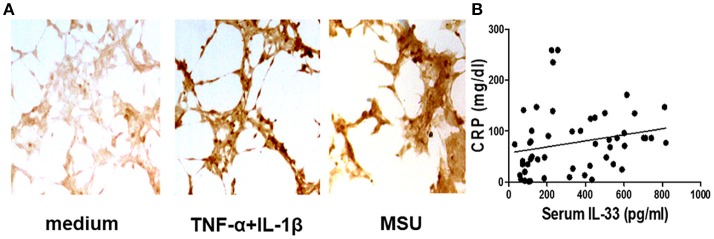
Corrrelation of the increased IL-33 with CRP in gout patients **(A)**. The synovial fibroblasts from synovial fluids were harvested to stimulate with TNF-α/IL-1β and MSU for 24 h, and then were stained with anti-IL-33 antibody by immunohistochemistry analysis. The results shown are from one of three independent experiments **(B)**. The sera collected from gout patients were used to analyze IL-33 levels by ELISA. The determination of linear relationships between IL-33 expression and CRP in gout patients was performed by Spearman correlation coefficient (*r* = 0.38, *p* = 0.005).

Our previous study has shown that the serum IL-33 level was predominantly increased in gout patients when compared to healthy controls, and the increased IL-33 expression might play a protective role in kidney injury by regulating lipid metabolism in gout (25). In this study, we recruited more participants to compare levels of IL-33 in gout patients and healthy volunteers. Consistent with our previous study, an increased expression of IL-33 was observed in the sera of gout patients compared with healthy control (data not shown). It has been reported that IL-33 was expressed in synovial fibroblasts from patients with rheumatoid arthritis (RA), and expression was markedly elevated *in vitro* by TNFα and IL-lβ stimulation (13, 26, 27). Deposition of MSU in the articular cavity can stimulate resident tissue macrophages to produce inflammatory factors TNFα and IL-lβ. Therefore, synovial fibroblasts from gout patients with gouty arthritis were separated and treated with MSU or TNFα/IL-lβ. Consistently, TNFα and IL-lβ also induced the up-regulation of IL-33 expression in the synovial fibroblasts from gout patients. In addition, we also found that MSU can directly induce the expression of IL-33 in synovial fibroblasts (Figure 1A). CRP was an acute time-phase reaction protein and the most common inflammatory marker for disease activity index in acute gout. Although a protective role of IL-33 in the kidney injury of gout was observed, we here found a positive correlation between the increased IL-33 expression and inflammatory indicator CRP (*r* = 0.38, *p* = 0.005; Figure 1B). Our data suggested that IL-33 might modulate MSU-induced inflammation.

### IL-33 Reduces the Development of Experimental Gout in Mice

**Figure 2:**
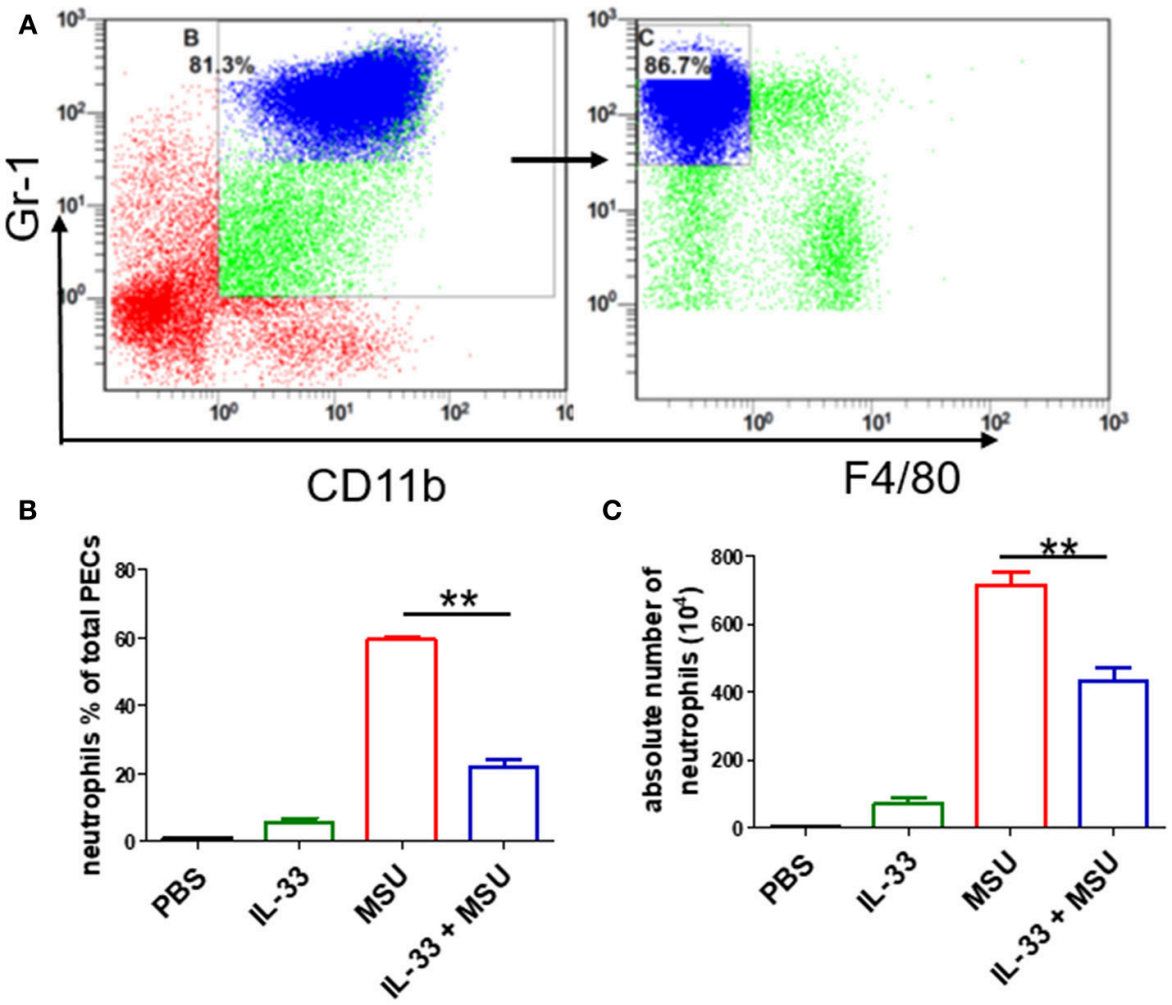
IL-33 reduces the neutrophils recruitment in gout animal model. Mice treated with IL-33 or PBS for 4 consecutive days, then injected with MSU or PBS. The mice were sacrificed after 16 h, and the cells in the peritoneal cavity were harvested and analyzed by flow cytometry **(A)**. Neutrophils are defined as cells with CD11b^+^Gr-1^+^F4/80^−^ surface marker **(B,C)**. The infiltrated inflammatory cells in the peritoneal cavity were analyzed by flow cytometry for neutrophils. Data represent mean ± SEM per group (*n* = 7/group). **p < 0.001.

Next, we sought to determine the role of increased expression of IL-33 in gout by using MSU-induced peritonitis experimental model. The exogenous IL-33 was given intraperitoneally daily before MSU treatment for 4 continuous days. The infiltrated leukocytes in the peritoneal cavity were harvested to analyze after MSU administration. Because neutrophils are the important effector cells in MSU-induced inflammation, the peritoneal exudate cells were subjected to analyze the neutrophils by flow cytometry. The CD11b^+^Gr-1^high^F4/80^−^ cells were considered as neutrophils (Figure 2A). The percentage of neutrophils in mice treated with PBS was very low, and exogenous IL-33 treatment slightly elevated the percentage of neutrophils. As expected, the percentage of neutrophils was significantly increased after MSU treatment. However, the percentage of neutrophils induced by MSU administration was significantly decreased in the mice with IL-33 treatment (Figure 2B). In addition, we also analyzed the absolute number of neutrophils in these mice. In keeping with the percentage, the absolute number of neutrophils in the MSU-treated mice was also significantly decreased in the mice with IL-33 administration (Figure 2C). Collectively, these results indicated that IL-33 can prevent the recruitment of neutrophils in MSU-induced acute inflammation.

### IL-33 Shapes the Cytokines Profiles in MSU-Induced Inflammation

**Figure 3:**
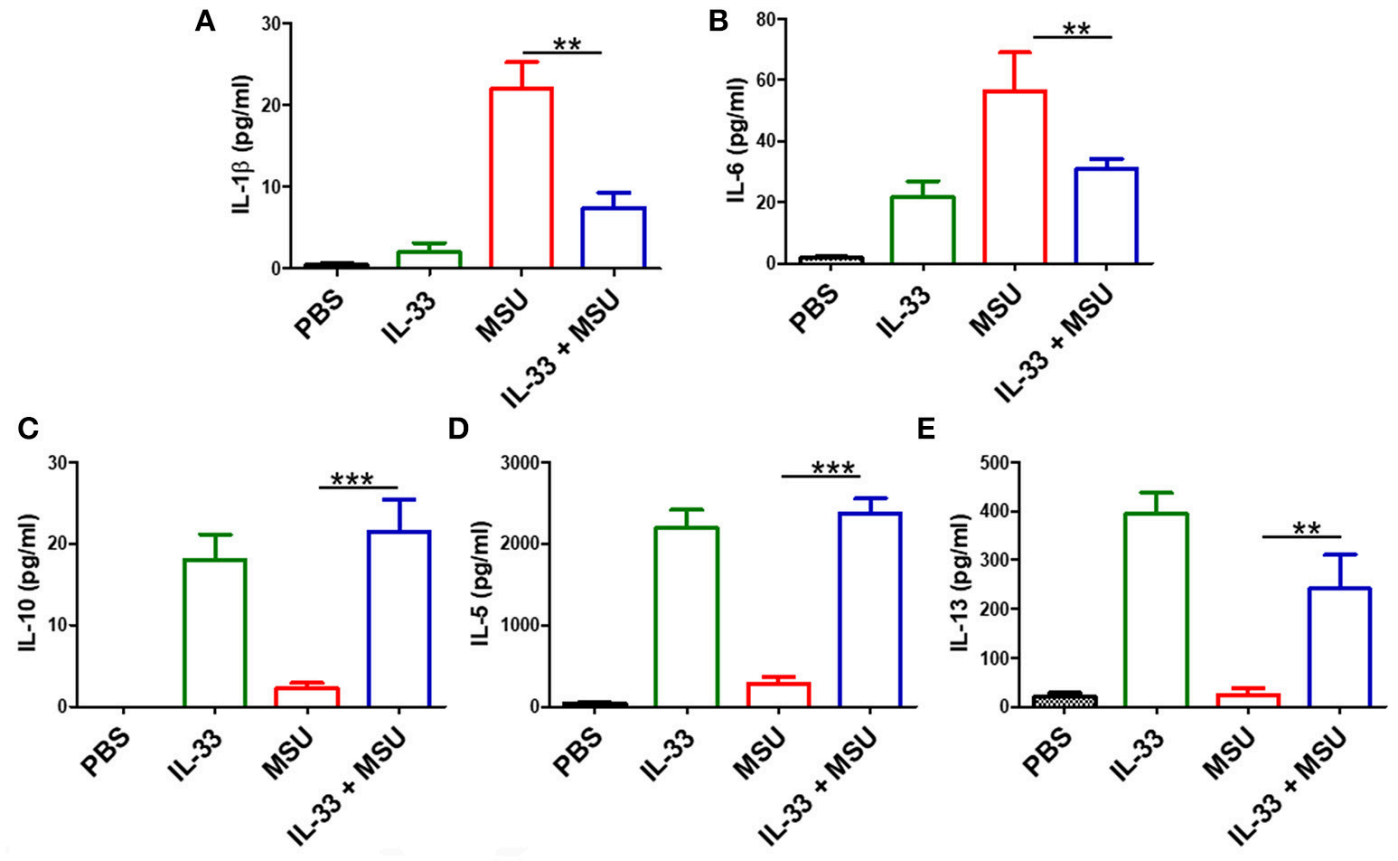
IL-33 administration affects cytokines profile in MSU-induced inflammation. The peritoneal cavity gavage fluids were harvested after MSU treatment 16 h, and performed to detect the IL-1β, IL-6, IL-10, IL-5, and IL-13 cytokines levels by ELISA **(A,B)**. The expression of IL-1β and IL-6 in the MSU-inflammation was decreased by IL-33 treatment **(C–E)**. The expressions of IL-10, IL-5, and IL-13 in mice after IL-33 and MSU pretreatment were also analyzed by ELISA. Data are shown as the mean ± SEM (*n* = 7/group) and are representative of three independent experiments; **p < 0.001, ***p < 0.0001.

The above data showed that IL-33 played a protective role in the development of gout, we here further explored whether exogenous IL-33-induced amelioration of MSU-induced inflammation correlated with reduction of IL-1β, which is the critical cytokine in the development of MSU-induced inflammation. The levels of IL-1β in the peritoneal cavity lavage were analyzed in our study. As expected, the expression of IL-1β was significantly decreased in the gout model mice with IL-33 treatment (Figure 3A). Besides, the inflammatory cytokine IL-6 was also inhibited by IL-33 treatment in the gout animal model (Figure 3B). IL-10 is an anti-inflammatory cytokine and plays a role in regulating inflammatory response. Here, we found that IL-33 administration evidently up-regulated the anti-inflammatory cytokine IL-10 production when compared with the PBS group (Figure 3C). In consistent with previous studies, we also observed an increased level of IL-5 and IL-13 in the peritoneal cavity after exogenous IL-33 treatment (Figures 3D,E). Taken together, IL-33 could up-regulate the expression of anti-inflammatory cytokines and inhibit the pro-inflammatory cytokines production in MSU-induced inflammation.

### Blockage of Endogenous IL-33 Signaling Has No Effect on MSU-Induced Inflammation

**Figure 4:**
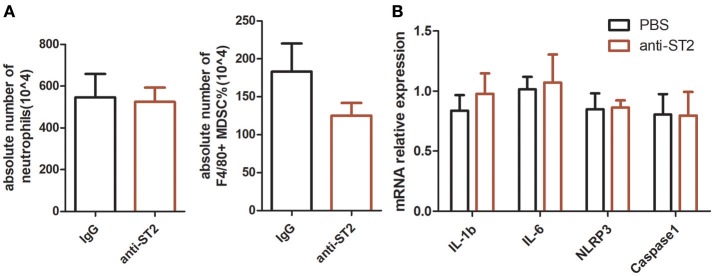
Inhibition of endogenous interleukin-33 signaling has no effect on the development of MSU-induced peritonitis. Mice were pretreated with a neutralizing anti-ST2 antibody or control IgG 1 h before the injection of MSU. The mice were sacrificed after 16 h of MSU treatment, and the peritoneal exudate cells were harvested. The peritoneal cells were stained with anti-CD11b, anti-Gr-1, and anti-F4/80 antibodies **(A)**. The absolute number of neutrophils and MDSCs were analyzed by performing flow cytometry. Data represent mean ± SEM per group (*n* = 5/group). *p* > 0.05 **(B)**. The RNA of peritoneal cells were harvest and performed to detect the IL-1β, IL-6, NLRP3, and caspase-1 mRNA expression by RT-PCR method. *p* > 0.05.

Exogenous recombinant IL-33 treatment significantly inhibited the development of MSU-induced inflammation. To investigate the functional effects of endogenous increased IL-33 production in MSU-induced inflammation, the endogenous IL-33 signaling was blocked by the administration of anti-ST2 antibodies. However, mice treated with anti-ST2 antibody were unaffected by the neutrophils infiltration and MDSCs expansion in the MSU-induced inflammation. Furthermore, the expressions of IL-1β, IL-6, NLRP3, Caspase-1 were detected by RT-PCR, while no significant difference was observed between control group and anti-ST2 group (Figure 4). Therefore, we speculate that an increased expression of IL-33 in the gout patients might be due to a cause of self-negative regulation, while the increased amount of IL-33 expression was inadequate to induce a potent protective effect to reduce the development of gout.

### IL-33 Inhibits MSU-Induced Inflammation Through Expanding MDSCs

**Figure 5:**
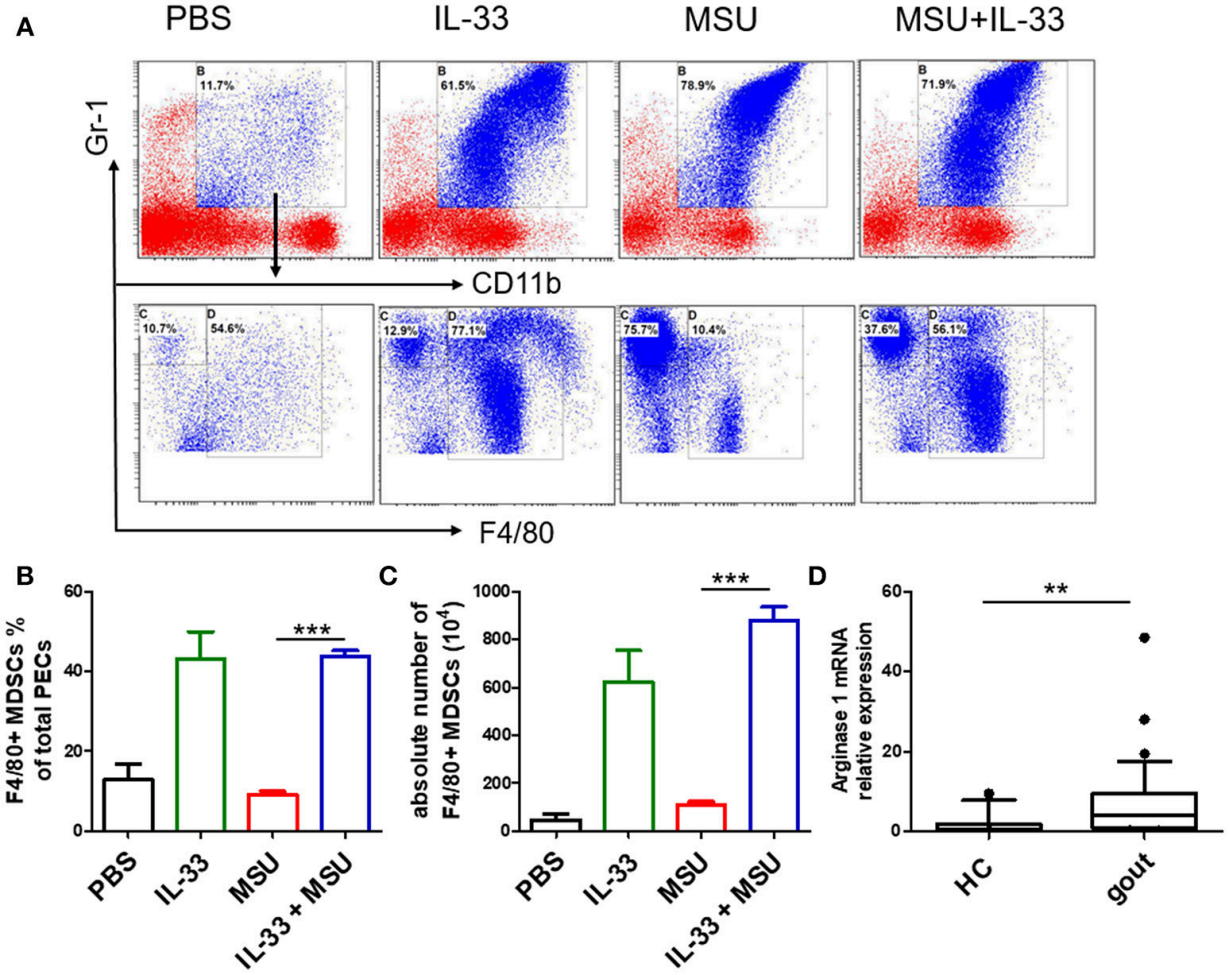
IL-33 promoted MDSCs expansion in gout animal model. Mice were pretreated with IL-33 or PBS for 4 consecutive days prior to injection of MSU. The mice were sacrificed after 16 h of MSU treatment, and the cells from the peritoneal cavity were harvested **(A)**. The peritoneal cells were stained with anti-CD11b, anti-Gr-1, and anti-F4/80 antibodies. CD11b^+^Gr-1^+^F4/80^−^ cells were considered to be neutrophils, while CD11b^+^Gr-1^+^F4/80^+^ cells represent MDSCs **(B)**. Data are presented as the percentage of CD11b^+^Gr-1^+^F4/80^+^ cells in CD11b^+^Gr-1^+^ cells **(C)**. The absolute number of CD11b^+^Gr-1^+^F4/80^+^ cell was also quantified. The results shown are from one of three independent experiments. ***p < 0.0001 **(D)**. The PBMCs collected from gout patients and healthy control (HC) were subjected to analyze the Arginase-1 expression by RT-PCR method. The Mann-Whitney *U* test was used to calculate the significance. **p < 0.001.

**Figure 6:**
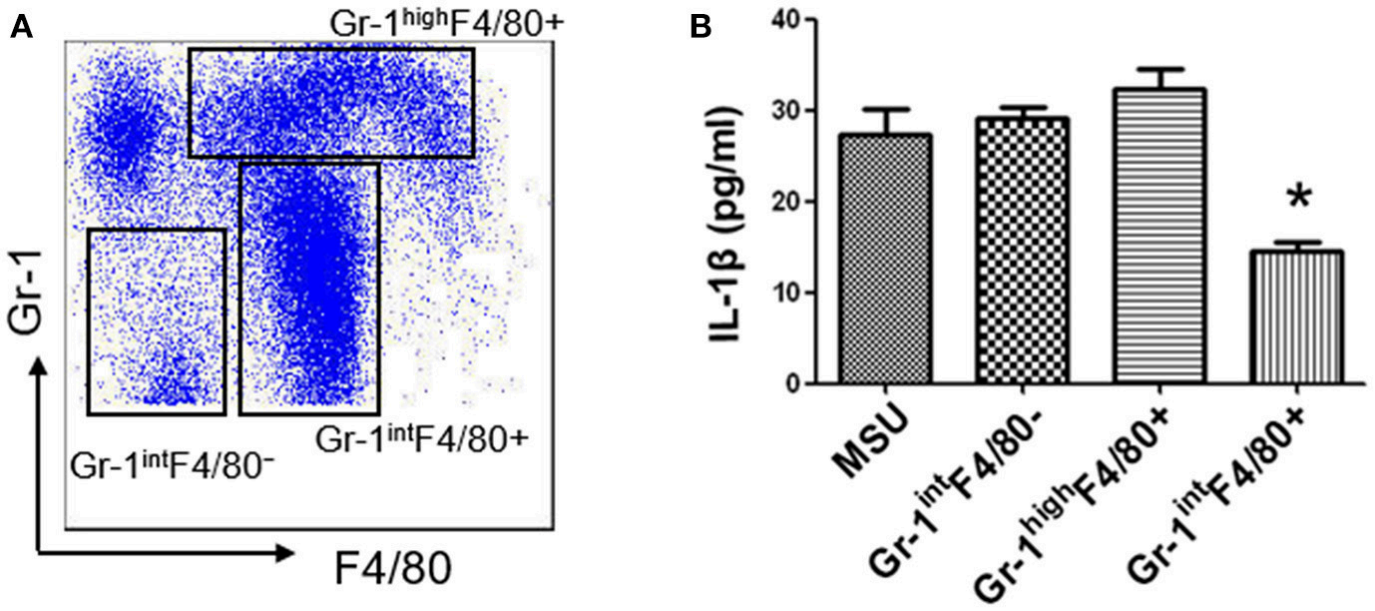
IL-33 suppresses IL-1β production through expanding CD11b^+^Gr-1^int^F4/80^+^ MDSCs **(A)**. Subpopulation (CD11b^+^Gr-1^high^F4/80^+^, CD11b^+^Gr-1^int^F4/80^+^, and CD11b^+^Gr-1^int^F4/80^−^) induced by IL-33 in wild type mice were sorted by flow cytometry. The dot plots were gated from CD11b^+^ cells **(B)**. Three groups of cells were successively adoptive transfer to the MSU-induced gout model mice. Four groups of mice (including a group of control mice treated with MSU only) were sacrificed and peritoneal lavage fluids were subjected to analyze the IL-1β production. Data are shown as the mean ± SEM (*n* = 7/group) and are representative of three independent experiments. *p < 0.05.

MDSCs are a group of heterogeneous cells, which consist of granular cell-like MDSCs and mononuclear cell-like MDSCs. By flow cytometry analysis of the collective peritoneal cells, we observed a large number of MDSCs in the PECs of the mice with IL-33 treatment. Specially, the increased number of MDSCs induced by IL-33 in the animal gout model was characterized by surface markers CD11b^+^Gr-1^int^F4/80^+^ (Figures 5A–C). Arginine1 was also recognized as an important marker for MDSCs, we detected the expression of arginine1 in the gout and healthy control PBMCs at the mRNA level by RT-PCR. We observed an increased expression of arginine1 in the gout patients when compared with healthy controls (Figure 5D). These results demonstrated that IL-33 can induce MDSCs recruitment, which might be involved in the self-limiting of the MSU-induced inflammation in the gout patients. Furthermore, three groups of cells induced by IL-33 were isolated and adoptively transferred to the gout model mice (Figure 6A). The expression levels of cytokines IL-1β in the peritoneal cavity were analyzed. Compared to other groups, the expression of IL-1β cytokine in the MSU-inflammation was decreased by CD11b^+^Gr-1^int^F4/80^+^ cells transfusion (Figure 6B). Thus, the CD11b^+^Gr-1^int^F4/80^+^ expansion induced by IL-33 exerts an important role in the resolution of MSU-induced inflammation.

## Discussion

In this study, we found an increased level of IL-33 in gout patients, which was positively correlated with inflammatory marker CRP. However, exogenous recombinant IL-33 significantly ameliorated the development of animal gout model. Interestingly, a large number of CD11b^+^Gr1^int^F4/80^+^ MDSCs was detected in the IL-33-treated mice, and adoptive transfer of IL-33-induced MDSCs markedly inhibited the IL-1β production in MSU-induced peritonitis. This is the first to document the role of IL-33 in a mouse model of human gout. Current results provide evidences for a novel mechanism by which IL-33 ameliorates the MSU-induced inflammation via expanding CD11b^+^Gr1^int^F4/80^+^ MDSCs.

IL-33 is a cytokine that is widely expressed in a variety of tissue cells, especially fibroblasts (8). Its production can be upregulated by inflammatory cytokines, such as such as IL-1β and TNF-α. Unlike conventional cytokines, IL-33 might be also secreted via unconventional pathways, and can be released upon cell injury as an alarmin. Here, we also observed an increased production of IL-33 in fibroblasts from gout patients, which were stimulated by inflammatory cytokines or MSU. Recent studies have shown that IL-33 played a complex effect in many diseases by interacting with its specific receptor ST2 (7). It has been reported that IL-33 plays a deleterious role in Th2-type immune mediated asthma and Th17-mediated autoimmune arthritis (11, 13). Conversely, a protective role of IL-33 was observed in atherosclerosis, sepsis, allograft transplant and parasite infection (9). Indeed, the IL-33/ST2 signaling also played a dichotomous role in inflammatory bowel disease pathogenesis (28). Our previous studies showed that IL-33 was markedly increased in the mice with TNBS-induced colitis, while rIL-33 treatment had a significant beneficial effect on Th1/Th17-mediated experimental colitis through promoting alternatively activated macrophage polarization and Tregs differentiation (24, 29). Interestingly, in our present study, although the level of IL-33 expression was increased in the gout patients and positively correlated with inflammatory marker CRP, the exogenous IL-33 treatment significantly inhibited the development of inflammation induced by MSU administration. In addition, blockage of IL-33 signaling by anti-ST2 antibody had no effect on the development of MSU-induced peritonitis. An interesting character of acute gout is the self-limiting nature of the inflammatory flare. In the absence of clinical intervention, a gouty episode can spontaneously resolve within 7–10 days. However, the mechanism of inflammation self-resolution is still elusive. Here, we speculate that an increased expression of IL-33 in the gout patients might due to a cause of self-negative regulation, which inhibits the development of MSU-induced inflammation, while the increased amount of IL-33 expression was inadequate to induce a potent protective effect to reduce the development of gout.

Acute gouty attacks are by nature self-limiting, and multiple mechanisms have been proposed for the spontaneous resolution of acute gout. Previous studies have shown that activated neutrophil impedes the interaction between immunoglobulin G (IgG) and MSU, leading to decrease the inflammation response. Besides that, ApoB and ApoE also exerts a key role in the resolution of acute gout through coating MSU crystal (30). Recently, the formation of neutrophil extracellular traps (NETs) has gained increased attention in self-limiting gout inflammation. NETs can neutralize bacteria as well as other danger signals such as MSU crystals by the rapid extrusion of DNA, and can cleave inflammatory cytokines within minutes (5). Numerous studies have showed that MDSCs play an important role in regulating immune responses through producing arginase I, IL-10, and TGF-β (16). In allogeneic transplantation and tumor environments, IL-33 could regulate the induction and migration of MDSCs to inhibit transplant rejection and promotes the progression of mouse breast cancer, the mechanism of which is related to its inhibition of T cell responses (31). Here, we observed a large number of MDSCs (CD11b^+^Gr1^int^F4/80^+^) in MSU-treated mice, which was different from neutrophil (CD11b^+^Gr1^high^F4/80^−^) phenotype. Furthermore, adoptive transfer of IL-33-induced MDSCs (CD11b^+^Gr1^int^F4/80^+^) markedly inhibited IL-1β production in MSU-induced peritonitis. Our study here presented a new clue for the self-limiting acute gout, and the increased IL-33 expression in gout patients might be a negative feedback mechanism on MSU-induced inflammation. Consistently, a recent study reported that LPS induces mononuclear cell-like MDSCs which can prevent tissue damage in acute lung injury through clearing apoptotic neutrophils (23). The clearance of apoptotic neutrophils by MDSCs was dependent on IL-10. In MSU crystal-induced inflammation, the IL-10 expression was increased after IL-33 treatment. We speculate that IL-33-induced MDSCs might alleviate the development of gout inflammation through phagocytosing neutrophils.

Our data demonstrated that increased IL-33 expression in gout patients might be a negative feedback mechanism on the MSU-induced inflammation response, because the inflammatory cytokine IL-1β production and the number of neutrophils were both markedly decreased in IL-33-treated mice. Furthermore, we reveal a requirement for MDSCs (CD11b^+^Gr1^int^F4/80^+^) in resolution of MSU-induced inflammation. In conclusion, our data provide clear evidences that the increased expression of IL-33 in gout patients was involved in the self-resolution of MSU-induced inflammation, and IL-33 might be a promising therapeutic target for gout.

## Author Contributions

KS, YYW, and LD analyzed data and wrote the manuscript. YYW, KS, QS, and BY performed the experiments. KS and YT contributed to the clinical patients' sample collection. YH provided technical support. KS, YLW, and GS provided critical revision of the manuscript. LD and GS designed the research and revised the article. All authors have read and approved the final manuscript.

### Conflict of Interest Statement

The authors declare that the research was conducted in the absence of any commercial or financial relationships that could be construed as a potential conflict of interest.

## References

[1] Richette P Bardin T. Gout. Lancet (2010) 375:318–28. 10.1016/S0140-6736(09)60883-7 19692116

[2] Choi HK Mount DB Reginato AM American College of Physicians, American Physiological Society. Pathogenesis of gout. Ann Intern Med. (2005) 143:499–516. 10.7326/0003-4819-143-7-200510040-00009 16204163

[3] Martinon F Petrilli V Mayor A Tardivel A Tschopp J. Gout-associated uric acid crystals activate the NALP3 inflammasome. Nature (2006) 440:237–41. 10.1038/nature04516 16407889

[4] Chen CJ Shi Y Hearn A Fitzgerald K Golenbock D Reed G. MyD88-dependent IL-1 receptor signaling is essential for gouty inflammation stimulated by monosodium urate crystals. J Clin Invest. (2006) 116:2262–71. 10.1172/JCI28075 16886064 PMC1523415

[5] Schett G Schauer C Hoffmann M Herrmann M. Why does the gout attack stop? A roadmap for the immune pathogenesis of gout. RMD Open (2015) 1(Suppl. 1):e000046. 10.1136/rmdopen-2015-000046 26557370 PMC4632150

[6] Cronstein BN Terkeltaub R. The inflammatory process of gout and its treatment. Arthritis Res Ther. (2006) 8 (Suppl. 1):S3. 10.1186/ar1908 16820042 PMC3226108

[7] Schmitz J Owyang A Oldham E Song Y Murphy E McClanahan TK. IL-33, an interleukin-1-like cytokine that signals via the IL-1 receptor-related protein ST2 and induces T helper type 2-associated cytokines. Immunity (2005) 23:479–90. 10.1016/j.immuni.2005.09.015 16286016

[8] Liew FY Pitman NI McInnes IB. Disease-associated functions of IL-33: the new kid in the IL-1 family. Nat Rev Immunol. (2010) 10:103–10. 10.1038/nri2692 20081870

[9] Liew FY. IL-33: a Janus cytokine. Ann Rheum Dis. (2012) 71 (Suppl. 2):i101–4. 10.1136/annrheumdis-2011-200589 22460136

[10] Arend WP Palmer G Gabay C. IL-1, IL-18, and IL-33 families of cytokines. Immunol Rev. (2008) 223:20–38. 10.1111/j.1600-065X.2008.00624.x 18613828

[11] Liu X Li M Wu Y Zhou Y Zeng L Huang T. Anti-IL-33 antibody treatment inhibits airway inflammation in a murine model of allergic asthma. Biochem Biophys Res Commun. (2009) 386:181–5. 10.1016/j.bbrc.2009.06.008 19508862

[12] Haenuki Y Matsushita K Futatsugi-Yumikura S Ishii KJ Kawagoe T Imoto Y. A critical role of IL-33 in experimental allergic rhinitis. J Allergy Clin Immunol. (2012) 130:184–94 e11. 10.1016/j.jaci.2012.02.013 22460070

[13] Palmer G Talabot-Ayer D Lamacchia C Toy D Seemayer CA Viatte S. Inhibition of interleukin-33 signaling attenuates the severity of experimental arthritis. Arthritis Rheum. (2009) 60:738–49. 10.1002/art.24305 19248109

[14] Yin H Li XY Jin XB Zhang BB Gong Q Yang H. IL-33 prolongs murine cardiac allograft survival through induction of TH2-type immune deviation. Transplantation (2010) 89:1189–97. 10.1097/TP.0b013e3181d720af 20220570

[15] Miller AM Xu D Asquith DL Denby L Li Y Sattar N. IL-33 reduces the development of atherosclerosis. J Exp Med. (2008) 205:339–46. 10.1084/jem.20071868 18268038 PMC2271006

[16] Gabrilovich DI Nagaraj S. Myeloid-derived suppressor cells as regulators of the immune system. Nat Rev Immunol. (2009) 9:162–74. 10.1038/nri2506 19197294 PMC2828349

[17] Highfill SL Rodriguez PC Zhou Q Goetz CA Koehn BH Veenstra R. Bone marrow myeloid-derived suppressor cells (MDSCs) inhibit graft-versus-host disease (GVHD) via an arginase-1-dependent mechanism that is up-regulated by interleukin-13. Blood (2010) 116:5738–47. 10.1182/blood-2010-06-287839 20807889 PMC3031417

[18] Zoso A Mazza EM Bicciato S Mandruzzato S Bronte V Serafini P. Human fibrocytic myeloid-derived suppressor cells express IDO and promote tolerance via Treg-cell expansion. Eur J Immunol. (2014) 44:3307–19. 10.1002/eji.201444522 25113564

[19] Marigo I Bosio E Solito S Mesa C Fernandez A Dolcetti L. Tumor-induced tolerance and immune suppression depend on the C/EBPbeta transcription factor. Immunity (2010) 32:790–802. 10.1016/j.immuni.2010.05.010 20605485

[20] Brunner SM Schiechl G Falk W Schlitt HJ Geissler EK Fichtner-Feigl S. Interleukin-33 prolongs allograft survival during chronic cardiac rejection. Transpl Int. (2011) 24:1027–39. 10.1111/j.1432-2277.2011.01306.x 21797940

[21] Lu B Yang M Wang Q. Interleukin-33 in tumorigenesis, tumor immune evasion, and cancer immunotherapy. J Mol Med. (2016) 94:535–43. 10.1007/s00109-016-1397-0 26922618

[22] Turnquist HR Zhao Z Rosborough BR Liu Q Castellaneta A Isse K. IL-33 expands suppressive CD11b+ Gr-1(int) and regulatory T cells, including ST2L+ Foxp3+ cells, and mediates regulatory T cell-dependent promotion of cardiac allograft survival. J Immunol. (2011) 187:4598–610. 10.4049/jimmunol.1100519 21949025 PMC3197898

[23] Poe SL Arora M Oriss TB Yarlagadda M Isse K Khare A. STAT1-regulated lung MDSC-like cells produce IL-10 and efferocytose apoptotic neutrophils with relevance in resolution of bacterial pneumonia. Mucosal Immunol. (2013) 6:189–99. 10.1038/mi.2012.62 22785228 PMC3505806

[24] Duan L Chen J Zhang H Yang H Zhu P Xiong A. Interleukin-33 ameliorates experimental colitis through promoting Th2/Foxp3(+) regulatory T-cell responses in mice. Mol Med. (2012) 18:753–61. 10.2119/molmed.2011.00428 22426954 PMC3409280

[25] Duan L Huang Y Su Q Lin Q Liu W Luo J. Potential of IL-33 for preventing the kidney injury via regulating the lipid metabolism in gout patients. J Diabetes Res. (2016) 2016:1028401. 10.1155/2016/1028401 27579324 PMC4992512

[26] Kunisch E Chakilam S Gandesiri M Kinne RW. IL-33 regulates TNF-alpha dependent effects in synovial fibroblasts. Int J Mol Med. (2012) 29:530–40. 10.3892/ijmm.2012.883 22246057 PMC3573710

[27] Xu D Jiang HR Kewin P Li Y Mu R Fraser AR. IL-33 exacerbates antigen-induced arthritis by activating mast cells. Proc Natl Acad Sci USA. (2008) 105:10913–8. 10.1073/pnas.0801898105 18667700 PMC2491487

[28] Hodzic Z Schill EM Bolock AM Good M. IL-33 and the intestine: the good, the bad, and the inflammatory. Cytokine (2017) 100:1–10. 10.1016/j.cyto.2017.06.017 28687373 PMC5650929

[29] Tu L Chen J Xu D Xie Z Yu B Tao Y. IL-33-induced alternatively activated macrophage attenuates the development of TNBS-induced colitis. Oncotarget (2017) 8:27704–14. 10.18632/oncotarget.15984 28423665 PMC5438602

[30] Scanu A Oliviero F Gruaz L Sfriso P Pozzuoli A Frezzato F. High-density lipoproteins downregulate CCL2 production in human fibroblast-like synoviocytes stimulated by urate crystals. Arthritis Res Ther. (2010) 12:R23. 10.1186/ar2930 20149224 PMC2875657

[31] Nagaraj S Gabrilovich DI. Myeloid-derived suppressor cells in human cancer. Cancer J. (2010) 16:348–53. 10.1097/PPO.0b013e3181eb3358 20693846

